# Shifting perspectives from “oncogenic” to oncofetal proteins; how these factors drive placental development

**DOI:** 10.1186/s12958-018-0421-3

**Published:** 2018-10-19

**Authors:** Rachel C. West, Gerrit J. Bouma, Quinton A. Winger

**Affiliations:** Department of Biomedical Sciences, Animal Reproduction and Biotechnology Laboratory, Colorado State University, 10290 Ridgegate Circle, Lone Tree, Fort Collins, CO 80124 USA

**Keywords:** Cell proliferation, Migration, Invasion, Angiogenesis, Genomic instability, Placenta, Placental insufficiency

## Abstract

Early human placental development strongly resembles carcinogenesis in otherwise healthy tissues. The progenitor cells of the placenta, the cytotrophoblast, rapidly proliferate to produce a sufficient number of cells to form an organ that will contribute to fetal development as early as the first trimester. The cytotrophoblast cells begin to differentiate, some towards the fused cells of the syncytiotrophoblast and some towards the highly invasive and migratory extravillous trophoblast. Invasion and migration of extravillous trophoblast cells mimics tumor metastasis. One key difference between cancer progression and placental development is the tight regulation of these oncogenes and oncogenic processes. Often, tumor suppressors and oncogenes work synergistically to regulate cell proliferation, differentiation, and invasion in a restrained manner compared to the uncontrollable growth in cancer. This review will compare and contrast the mechanisms that drive both cancer progression and placental development. Specifically, this review will focus on the molecular mechanisms that promote cell proliferation, evasion of apoptosis, cell invasion, and angiogenesis.

## Background

During pregnancy, the female body undergoes incredible anatomic, metabolic, and physiological changes in the process of providing for the needs of a developing fetus. One of the most essential developments is the genesis of a placenta, which is critical for hormone production and gas and nutrient exchange between the mother and the fetus [1–3]. Any aberration in these physiological processes can cause devastating placental pathologies like preeclampsia and intrauterine growth restriction (IUGR) [4], leading to severe pregnancy complications [5]. Preeclampsia affects 4–8% of pregnancies in the United States and is attributed as the cause behind 500,000 fetal and 75,000 maternal deaths each year [6, 7]. IUGR also affects 7–9% of newborn infants and is thought to cause up to 50% of unexplained stillbirths [8]. These pregnancy complications can also cause long-term developmental delays and health consequences including; cerebral palsy, deafness, chronic lung disease, neurodevelopmental delays, and metabolic disorders [9–11], leading to substantial health care costs and emotional burdens on families. Both preeclampsia and IUGR appear to be heritable as they both are associated with an increased likelihood of IUGR and fetal death in subsequent pregnancies of the affected mothers [11]. Additionally, IUGR often occurs frequently in women suffering from placental morbidities such as preeclampsia, and gestational diabetes, putting the mother’s life in significant danger as well as the fetus [12].

The conditions affecting fetal growth can either be placental or fetal in origin. Fetal growth is dependent upon the overall health of the fetus, the ability of the mother to metabolize and provide sufficient amounts of substrates necessary for growth, and the competency of the placenta to transport these substrates from the mother to the fetus [13]. However, impaired placental function seems to drive the most severe cases of IUGR [14]. This placental insufficiency is a common phenotype associated with both IUGR and maternal placental comorbidities including preeclampsia and hypertension [15]. Currently, treatments for pathologies caused by placental insufficiency are lacking, with no known treatment for pre-eclampsia other than the immediate delivery of the fetus.

While the understanding of the consequences of IUGR and preeclampsia has increased exponentially over the past few decades, there is still a need to elucidate the underlying cause behind placental insufficiency during development. Understanding what is driving placental insufficiency during early development will be essential in the development of better diagnostic and treatment tools for the prevention and treatment of both pathologies. The delicate interplay between cell proliferation and differentiation could be a key event that malfunctions early on in pregnancy, eventually leading to placental dysfunction.

Typically, when one considers oncogenes it’s hard to ignore the profound effects these proteins have during normal homeostasis in adult tissues. These genes promote rampant cell proliferation in otherwise healthy tissues. Proliferative cells eventually begin to migrate towards other organ systems, invading into tissues to form metastatic tumors. However, to only consider oncogenes as “bad” fails to consider the original purposes of these genes. These oncogenic processes are essential during early embryonic, fetal, and placental development and any aberrant signaling by these genes can cause devastating effects on fetal growth. These proteins are responsible for the cancer-like processes that characterize early placental development. However, in direct contrast to carcinogenesis, the placenta uses these factors in a tightly controlled, highly regulated environment. This regulation exploits these factors so that they create a remarkably efficient organ in a short amount of time without the adverse consequences that often come with the expression of oncogenic proteins. Therefore, we propose that oncogenes instead be considered as oncofetal proteins.

This review will focus on the similarities of oncogenic processes like proliferation, escape of apoptosis, cell invasion and migration, angiogenesis, and the signaling pathways that drive these mechanisms in both cancer and placental development. Understanding these parallels between placentation and tumorigenesis will provide insight into not only better ways to treat cancer but also understand how these processes can fail during development leading to placental insufficiency.

### Human placental development

Placentation begins with the uterine endometrium changing its structure to prepare for implantation, a process known as decidualization [16]. The fibroblast-like cells of the endometrium transform into secretory decidual cells. These decidual cells comprise an immunoprivileged matrix that protects the implanting embryo from attack by maternal immune cells [17]. It also secretes the histotroph, an endometrial secretion that facilitates implantation and conceptus development during the initial weeks of pregnancy [18]. The histotroph also contains factors that regulate the invasion potential of the early trophoblast cells if an embryo implants [19].

**Fig. 1:**
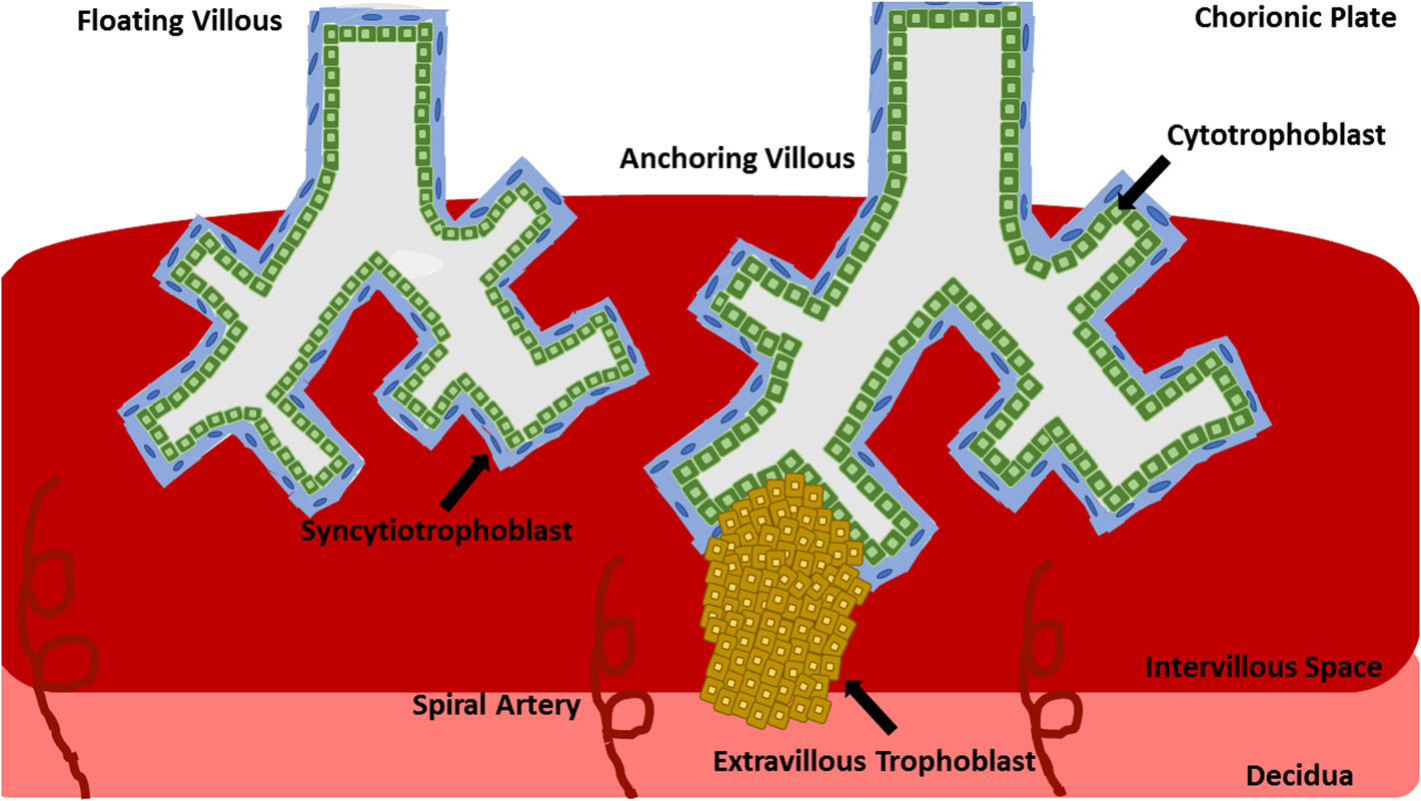
Early Placental Development. The progenitor cells of the placenta, the cytotrophoblast proliferate rapidly during the first trimester of pregnancy. During this time they also differentiate to become part of the syncytiotrophoblast layer that fuses and becomes the layer of the placenta that comes into contact with the maternal blood. Additionally, cytotrophoblast cells differentiate to become part of the extravillous trophoblast, the cells that invade into the mother’s endometrium, seeking out her spiral arteries

Once fertilization occurs, the zygote travels from the ampulla of the Fallopian tube to enter the endometrial cavity within 3 days [20]. During this journey, the zygote divides and undergoes a series of mitotic divisions to become the morula [21]. Approximately 5 days after fertilization, the morula transforms into a newly expanded blastocyst of 58-cells partitioned into a peripheral layer called the trophectoderm, that will eventually become the placenta and the inner cell mass (ICM), which will become the fetus [22]. Approximately 9 days after fertilization, the blastocyst implants into the uterine wall in a three step process called apposition, adhesion, and invasion [23]. At this timepoint, a multinucleated, primitive syncytium has formed, penetrating the decidua, hollowing out areas of the stromal layer, and forming the lacunae that will eventually be filled with maternal blood [24]. Additionally, by day 9 the progenitor trophoblast cells, cytotrophoblast cells, have begun to form villous structures that will eventually differentiate into the two main cell types of the placenta; the weakly proliferative and fusional syncytiotrophoblast and the terminally differentiated, invasive extravillous trophoblast (EVT) [25]. At day 12 of gestation, cytotrophoblast cells begin to penetrate the primitive syncytium, forming the first primary chorionic villi of the placenta [26]. The cytotrophoblast cells proliferate rapidly and accumulate in floating villi which will differentiate to form the syncytium. This layer of cells will eventually come into contact with the maternal blood [27]. Alternatively, cytotrophoblast cells will also form anchoring villi that will eventually attach to and invade into the mother’s decidualized endometrium, myometrium, and eventually her spiral arterioles [28] (Fig. 1). This balance between cytotrophoblast cell proliferation and subsequent differentiation into the invasive and migratory EVT has a marked similarity to how cancer cells form tumors and metastasize.

### Cell proliferation

As the placenta begins forming 1 week after fertilization and must begin to facilitate nutrient and gas exchange by the end of the first trimester, rapid and substantial cell proliferation is essential. However, unlike cancer, this cell proliferation is tightly regulated and cells lose their proliferative capacity once they undergo differentiation into the invasive EVT lineage. One group of genes that are responsible for cytotrophoblast cell proliferation are growth factors and their receptors [29]. Epidermal growth factor (EGF), hepatocyte growth factor (HGF), vascular endothelial growth factor (VEGF), and placental growth factor (PLGF), insulin like growth factor (IGF), transforming growth factor (TGF) and their subsequent receptors have all been identified in the cytotrophoblast and are speculated to act in a paracrine and autocrine manner on the differentiated cells of the placenta [30–36]. These growth factors bind to tyrosine kinase receptors on cytotrophoblast cell membranes inducing self-dimerization to activate the MEK/ERK proliferation pathway and the PI3K/Akt anti-apoptosis pathway [37]. These kinase signaling cascades are potent catalysts that influence cell proliferation and survival in many cell types, including the placenta [38]. Gene editing experiments targeting the MAPK pathway in mice was embryonic lethal by E11.5 due to severe placental defects [39]. Additionally, gene disruption of the PI3K/Akt pathway led to depleted cells in the spongiotrophoblast layer (cells of the junctional zone of the mouse placenta, the specific function is still unclear [40]) and decreased vascularization [41]. These data indicate a necessary role for growth factor driven activation of the MAPK/PI3K pathways during early placental development. Interestingly, the phosphorylated forms of ERK1 and ERK2 were only detected in proliferative cytotrophoblast cells until the end of the first trimester. This alludes to their importance in cell proliferation, losing expression once cells begin to terminally differentiate [42].

Additional oncogenic downstream target of the MAPK pathway, JUN has also been implicated in early placental cell proliferation and differentiation. However, different members of the JUN family are expressed at different time points. Messenger RNA for *c-Jun* was found at its highest levels in early gestational placental tissue whereas *jun-B* was at its highest levels between 35 and 40 weeks [43]. The authors of this study concluded that in the placenta *c-jun* is essential for cytotrophoblast cell proliferation while *jun-B* likely plays a role in terminal differentiation. This conclusion is at least partially supported by another finding using stimulation by epidermal growth factor (EGF) to induce differentiation of human primary cytotrophoblast cells towards the syncytiotrophoblast fate. Cells were treated with EGF for 40 min pulses and, while both c-jun and jun-B mRNA levels rapidly increased 2–4 h after exposure, EGF’s effects on jun-B were the most striking. Jun-B was significantly increased in cytotrophoblast cells differentiating towards the syncytiotrophoblast lineage, indicating that EGF and its activation of jun-B is important in the terminal differentiation of cytotrophoblast cells [44]. Interestingly, the hormone adiponectin has also been implicated as an important regulator for the JUN kinase pathway, with a particular emphasis on c-jun regulation. In normal placentas, adiponectin has an antiproliferative effect. However, in gestation diabetes mellitus (GDM) placentas, adiponectin levels are decreased with an increase in cell proliferation, potentially thought to be a contributor to the macrosomia seen in GDM babies. To test whether adiponectin actually inhibits c-Jun in GDM placentas, the choriocarcinoma cell line, BeWo, was treated with high levels of glucose. These high glucose treated cells had significantly lower levels of adiponectin, leading to increased c-Jun protein and increased cell proliferation. Furthermore, addition of adiponectin to high glucose treated cells inhibited c-Jun activation, suppressing cell proliferation [45].

There are also several oncofetal proteins outside of the family of growth factors that promote cell proliferation. For example, our laboratory studies the LIN28-let7-HMGA2 molecular axis. LIN28 is an RNA binding protein considered to be a key molecular factor that regulates the transition from a pluripotent, highly proliferative state to a terminally differentiated cell [46]. One of the main targets of LIN28 is the let-7 family of miRNAs. When cells are highly proliferative, LIN28 negatively regulates the let-7 family. However, as cells begin to differentiate the let-7 family of miRNAs is upregulated and can bind to the 3’ UTR of *LIN28* to inhibit its translation into protein [47]. Because of this negative feedback loop, LIN28 and the let-7 s are often inversely expressed in many cancers [48]. In addition to this, increased LIN28 has been correlated with highly aggressive cancers and poor prognosis [49]. The let-7 s also regulate several other oncofetal proteins including HMGA2, c-Myc, RAS, and VEGF [49]. In placental cells, a knockdown of LIN28A led to spontaneous differentiation and syncytialization in human trophoblast cells [50]. Furthermore, knockdown of LIN28B and knockout of both LIN28A and LIN28B leads to trophoblast cells that are driven to differentiate towards only the syncytiotrophoblast lineage, but not extravillous trophoblast cells [51]. Collectively these data suggest that, as with pluripotent cells, LIN28 is an essential gatekeeper in trophoblast cell proliferation and differentiation.

### Cell survival

The ability to bypass apoptosis is another hallmark of cancer and is essential during placentation. Again, the growth receptors and receptor tyrosine kinase pathways mentioned above play an important role in cell survival, specifically IGF-1 and IGF-2 binding to IGF-1R [38, 52].The relationship between IGF-1R and the PI3K/Akt and MAPK pathways has been described as a crucial cell protectant in many different cancer cell types [53–56]. In immortalized human placental BeWo cells and in placental tissue explants both IGF1 and IGF2 rescued serum-starved cells from apoptosis [57]. Additionally, mutated IGF1-R in pregnant women leads to both intrauterine and post-natal growth restriction [58] and there is a direct correlation between IGF levels and birth weight [59].

There are two distinct mechanisms the IGF system targets to promote cell survival; the Bcl-2 family and caspase proteins [60]. Increased Bcl-2 expression has been reported in several cancer cell lines and tumors [61–64] and leads to increased cell survival and resistance to chemotherapy treatment [65]. Bcl-2 immunolocalization in the placenta has been described in several papers [66–68]; however its involvement in trophoblast cell apoptosis is still unclear. Soni et al. describe a gradual increase in Bcl-2 expression throughout pregnancy with maximal immunoreactivity occurring at term [69]. Ishihara et al. also suggest that based on their findings that abundant expression of Bcl-2 in term syncytiotrophoblast prevents cell death, allowing for the maintenance of placental mass near the end of pregnancy [66]. Additionally, the IGFs regulate caspase expression. Activation of IGF1-R can prevent cleavage of caspases in both cancer cells and fetal brain cells, preventing apoptosis [70, 71]. In accordance with the findings of Bcl-2 expression, there appears to be no caspase-mediated apoptosis in the syncytiotrophoblast of term villi of the placenta. There was also no response to stimulus-induced apoptosis in syncytiotrophoblast of villous explants from term placental tissue [72]. These data suggest that the syncytiotrophoblast can protect itself against apoptotic signals to continue to function and contribute to fetal growth until the end of pregnancy.

**Fig. 2:**
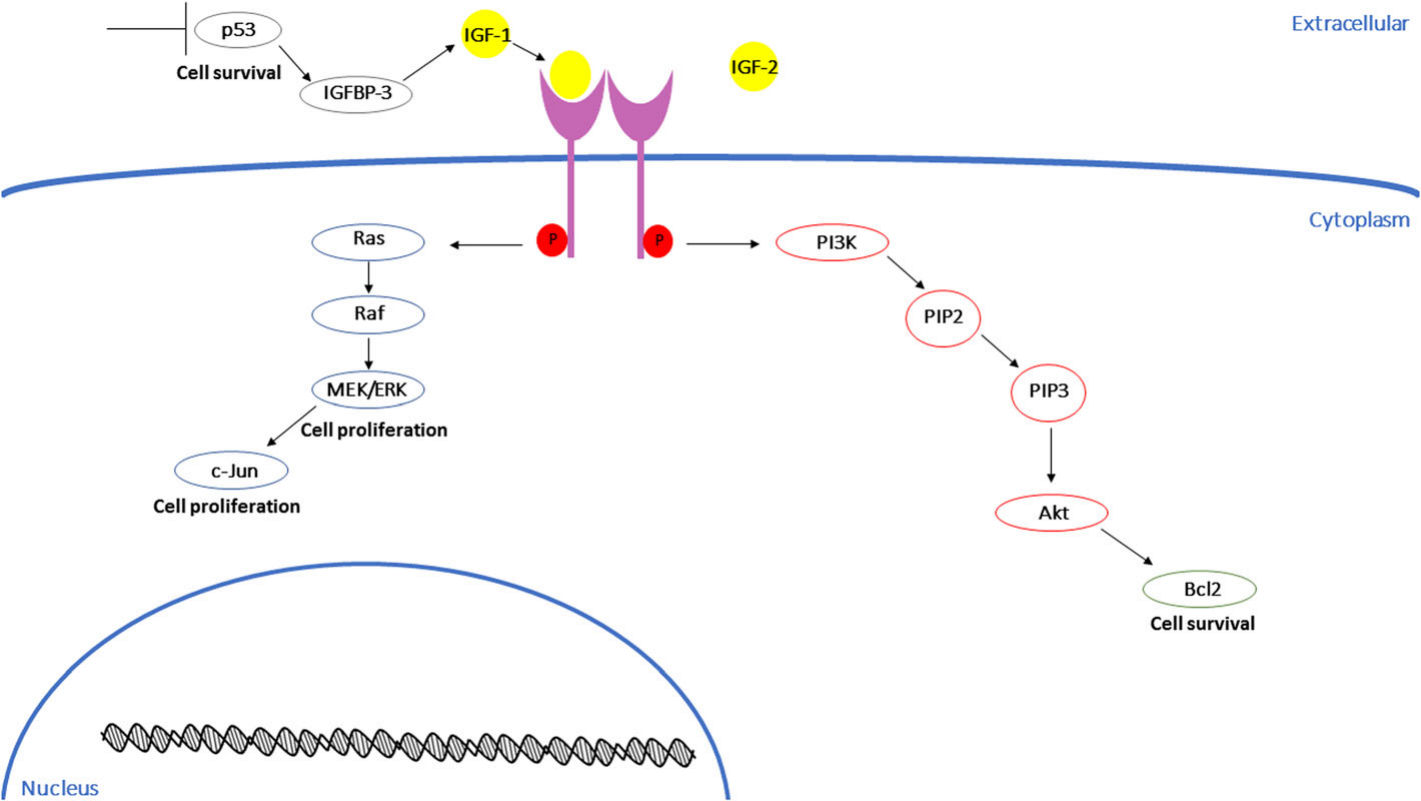
IGF signaling in the placenta. IGF regulates cell proliferation and survival in placenta cells through several mechanisms. Both IGF-1 and IGF-2 bind to the IGF-1R to stimulate the MEK/ERK pathway and the PI3K pathway to promote cell proliferation and evasion of apoptosis. Additionally, downregulation of p53 leads to higher levels of IGF’s allowing for more proliferation and cell survival

In most cell types, the transcription factor p53 antagonizes IGF signaling to promote apoptosis and cell cycle arrest [73]. Several papers report that p53 closely monitors the IGF-1/Akt pathway and, upon sensing stress, negatively regulates IGF-1/Akt to halt cell proliferation and induce autophagy [74–76]. This negative regulation occurs by p53 transactivating IGF-BP3. The family of IGF-BPs regulates ligand availability to their IGF receptors [77]. It has been shown that a p53-induced accumulation of IGF-BP3 in the extracellular medium of cells can inhibit mitogenic function of IGF-1 in vitro [78]. Increased IGF-BP3 leads to increased complexing to IGF-1, reducing their ability to bind IGF-1R to promote cell survival and proliferation [79]. However, over 50% of human cancers have p53 mutations, preventing it’s pro-apoptotic function to promote spontaneous tumorigenesis [80]. In the placenta, increased p53 protein expression in placental villi is correlated with pre-eclampsia [81]. As excessive apoptosis in the villous trophoblast of placental villi is a characteristic of pre-eclampsia, these data suggest that upregulated p53 induces a disproportionate amount of apoptosis, leading to placental insufficiency (Fig. 2).

Finally, another important anti-apoptotic factor often found in cancer is survivin [82]. Belonging to the “inhibitor of apoptosis” family, upregulation of survivin in cancers is directly correlated with apoptotic resistance, increased cell survival, and poor response to chemotherapy [83]. Survivin is yet another anti-apoptotic protein increased by IGF-1. In prostate cancer cells, stimulation with IGF-1 lead to increased survivin expression due to the increased stabilization and translation of survivin mRNA [84]. Alternatively, survivin has also been described as negatively regulated at the transcriptional level by p53 with the surivivin promoter having a p53 binding element although the exact mechanism of regulation by p53 is still poorly understood [82]. In the placenta, survivin is thought to play a crucial role in cell survival and proliferation of trophoblast cells [85, 86]. Messenger RNA levels of survivin were analyzed in first, second, and third trimester placentas of pre-eclamptic women, compared to normal placentas, survivin was significantly decreased. Additionally, survivin levels were directly correlated with severity of pre-eclampsia, with levels decreasing as pre-eclampsia became more severe [87]. Due to the upregulated levels of p53 in pre-eclampsia it has been suggested that the negative regulation of survivin by p53 is a potential cause of the low levels of survivin mRNA found in pre-eclamptic placentas [86].

### Cell invasion

Human placentation is unique in that the EVT cells of the placenta invade fully into the maternal decidua to encapsulate and erode the spiral arteries, exposing the placenta to maternal blood [88]. The similarities between cell invasion of EVT cells and cancer cells are striking. However, one key difference is that trophoblast cells adhere to a tightly regulated pattern of proliferation then differentiation and invasion without metastasis into new tissues. Cancer cells proliferate rapidly, eventually seeking out other tissues to metastasize towards. Not surprisingly, many of the same factors are required for both neoplastic cells and trophoblast cells. Some of these requirements for invasion include altered expression of cell adhesion molecules, secretion of proteinases, and epithelial-mesenchymal transition.

In non-invasive cells, there is a network of proteins that harness cells to the extracellular matrix (ECM) and to each other. However, in invasive or metastatic cells, this network is downregulated [89] which allows cells to seek out new tissues. One group of altered proteins is the integrin family. Integrins are a heterodimeric family of cell membrane proteins that are made up of at least 18 α subunits and 8 β subunits [90]. These subunits dimerize to form at least 24 different receptors, allowing them to bind to a variety of different ECM ligands. Because of this diversity, some integrins promote adhesion and some promote invasion. This review will only focus on the integrins that regulate cell invasion in the placenta.

During placental development, there is a delicate balance between adhesion-promoting integrin expression and invasion-promoting integrins. This balance in early cytotrophoblast cells is regulated in large part by α5β1 and α1β1. In contrast to cancer, cytotrophoblast cells use the invasion-restraining role of α5β1 to balance the invasion-promoting role of α1β1 to tightly regulate the depth of invasion into the mother’s decidua [91]. During early gestation, the proliferating cytotrophoblast cells begin to upregulate α1β1 as they differentiate to become more invasive. However, as gestation continues and invasion becomes less of a priority, expression of the α1β1 integrin complex declines [91]. Additionally in pre-eclamptic placental tissue, α1β1 immunostaining is almost nonexistent while the invasion-restraining α5β1 is still detectable at levels similar to normotensive placentas [92]. This suggests that the shallow invasion of uterine vasculature, a hallmark of pre-eclampsia, is at least in part caused by altered integrin expression.

The integrin family is inextricably linked with the TGF- β signaling pathway. TGF- β is both a regulator and regulated by several integrins in many different cell types [93]. Both α1β1 and α5β1 expression is stimulated by TGF- β in fibroblast cells. Additionally, α5β1 has been found to be upregulated by TGF- β in both carcinoma cells and lung cancer cells [93, 94]. As TGF- β is known to have an important role in both the inhibition and promotion of trophoblast cell invasion [95, 96], these data imply that there is a delicate interplay between TGF- β signaling and the regulation of the integrins α5β1 and α1β1 during early placental development.

Another driver of cell invasion shared between cancer and placentation is the loss of expression of the cell adhesion molecule E-cadherin. Found at the adherens junctions of epithelial cells, E-cadherin is a potent promoter of cell-cell adhesion [97]. Known as a suppressor of invasion, decreased function of E-cadherin is directly correlated with invasion and tumor metastasis [98, 99]. E-cadherin also plays a critical role in the maintenance of the epithelial cell phenotype, with a loss of E-cadherin being the final step to trigger the epithelial-mesenchymal transition (EMT) [100], a process that is not only important during early embryonic development but also cancer. E-cadherin is predominantly expressed in anchored placental villi of first and second trimester placentas, gradually becoming down-regulated as cells differentiate to become EVT [101]. The transcription factor Snail, transcriptionally regulates E-cadherin, by binding to the E-box elements found on Snail’s promoter region to trigger EMT and has also been suggested to regulate E-cadherin expression in EVT [102]. There is a layer of proliferative, non-invasive EVT cells found in the proximal and distal parts of anchored villi and as these cells undergo EMT to become invasive and migratory, there is a change in E-cadherin expression. However, term placentas from women with HELLP syndrome and pre-eclampsia found a reduction of E-cadherin in EVT cells with an apparent increase in Snail expression [102]. Snail appears to be the main regulator of decreased E-cadherin in most tumor expression and it now appears to be an important regulator of E-cadherin in EVT cells as well. Addtionally, E-cadherin is known to be essential for early embryonic and placental development as E-cadherin ^−/−^ mice have severe epithelial trophoblast defects and die at the time of implantation [103].

Finally, the metalloproteinase (MMP) family of proteins is a critical group of enzymes that facilitate invasion. In addition to degrading the ECM, MMPs also can modify cell adhesion molecules like integrins and activate cytokines to stimulate epithelial-mesenchymal transition and drive cell invasion [104]. Several MMPs, including MMP-2, MMP-3, and MMP-9 have been described in different locations in the placenta; however there is evidence to suggest that MMP-9 is the most influential proteinase during placental invasion [105, 106]. MMP-2 and MMP-9 are found at their highest levels in the extravillous cytotrophoblast between 6 and 8 weeks of pregnancy, appearing to facilitate trophoblast invasion into the decidua [107]. Interestingly, MMP expression isn’t restricted to the invasive trophoblast cells as MMPs have been described in the endometrial stromal and natural killer cells of the decidua [108]. Furthermore, permissiveness to invasion by the decidua seems to be influenced by the presence of cytotrophoblast cells. This interaction between uterine and trophoblast MMPs could be regulated by the pregnancy hormone, human chorionic gonadotropin (hCG). To stimulate maternal recognition of pregnancy during the first trimester, the developing embryo secretes proteins to decidualized endometrial stromal cells, allowing for upregulation of MMPs [109]. In immortalized JEG-3 cells and in villous tissue explants, addition of hCG to culture medium increased invasion in a dose dependent manner [110, 111]. Interestingly, these data suggest that the uterus has the ability to influence invasion, keeping this process regulated and local. This is in direct contrast to the unregulated and rampant invasion seen in metastatic cancer.

### Angiogenesis

**Fig. 3:**
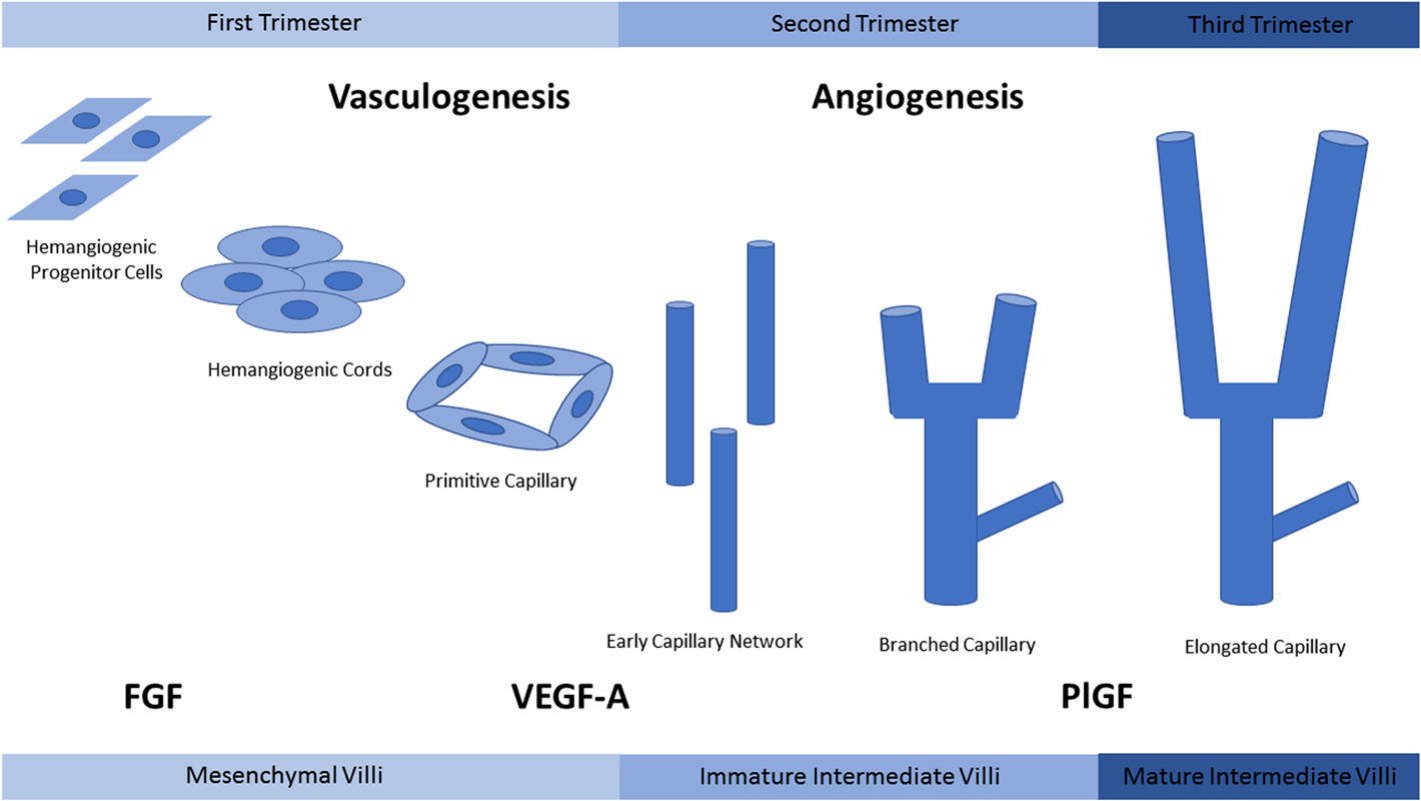
Angiogenesis in the placenta. During the first trimester FGF promotes vasculogenesis by promoting the differentiation of mesenchymal stem cells into hemangiogenic progenitor cells. These cells aggregate to form hemangiogenic cords and eventually primitive capillaries. VEGF-A promotes the angiogenesis of these capillaries through branching angiogenesis. As pregnancy progresses, PlGF is upregulated leading to non-branching angiogenesis and elongated capillaries

Angiogenesis is a mandatory process driving tumor pathogenesis leading to tumor metastasis and poor cancer prognosis. Alternatively, the ability to not only join existing vessels but also to create vessels in avascular tissue is an essential component of placental development. Any aberration in the signaling pathways that drive angiogenesis and vasculogenesis can lead to shallow invasion into the maternal spiral arteries, a known cause of placental insufficiency. The angiopoietin (ANG) and vascular endothelial growth factor (VEGF) families of growth factors are two critical families for vessel development in the placenta [112]. Similar to the balancing and counterbalancing effects of integrins regulating cell invasion, VEGF and placenta growth factor (PlGF) work in a synergistic fashion to promote angiogenesis in a controlled environment [113]. Both growth factors are key components that control two different types of angiogenesis, branching and non-branching. (Fig. 3).

Vasculogenesis begins approximately at 21 days post-conception when mesenchymal stem cells inside the mesenchymal villi of the placenta differentiate to become hemangiogenic progenitor cells [114]. These progenitor cells eventually migrate towards the periphery of the villous columns and coalesce to form hemangiogenic cords, the primitive original vessels of the villous [115]. Eventually these cords will mature into a more sophisticated network of vessels, differentiating into intermediate villi with capillary networks of branched vessels [116]. This process is almost totally driven by paracrine signaling of VEGF-A from the cytotrophoblast [114]. VEGF-A works through receptor tyrosine kinase receptors, VEGFR-1 and VEGFR-2, to stimulate branched angiogenesis [117]. Branching angiogenesis requires a series of steps including permeabilization of vascular tissue, degradation of the basement membrane, and increased proliferation and migration of endothelial cells. This leads to the formation of endothelial cell tubes and recruitment of pericytes to the exterior of the capillary, forming a stable vessel [115, 118]. These mechanisms lead to the creation of a network of immature intermediate villi containing superficially located capillaries lying directly beneath the trophoblast layer of the villous surface [119]. These branched vessels are responsible for the dramatic increase in villous blood vessels facilitating enhanced fetoplacental blood flow to accommodate the rapidly developing fetus [120]. Branching angiogenesis and VEGF-A expression continues to dominate placental vascularization quickly producing a multitude of vessels until approximately the 26th week of gestation [121]. At this point, villous vascularization undergoes a switch from branching to non-branching angiogenesis. At this point, the focus moves from producing more vessels to increasing the length of the existing vessels [122].

Non-branching angiogenesis is driven by another member of the VEGF family of proteins, PlGF. Whereas VEGF-A and VEGFR-2 are expressed at high levels during early pregnancy, waning as pregnancy advances [122]; PlGF is expressed at relatively low levels during the first trimester of pregnancy but increases at 11–12 weeks, reaching peak levels at week 30 of pregnancy [123]. PlGF is thought to have an antagonistic effect on VEGF-A, forming a heterodimer that prevents VEGF-A from activating either VEGF1-R or VEGF2-R [124]. At peak PlGF expression, the immature intermediate villi begin to form the mature intermediate villi. Non-branching angiogenesis leads to the formation of long, thin vessels found at the tips of the villous. These vessels continue to grow in length, eventually surpassing the boundaries of the mature intermediate villi to form terminal villi. Each terminal villous has a thin trophoblast layer covering only one or two capillary coils [125]. These villous structures are critical for diffusional gas exchange from mother to fetus [121] (Fig. 2).

Similarly to cancer, both VEGF and PlGF are regulated by hypoxia. In tumors, hypoxia has been shown to upregulate both VEGF and VEGFR expression [126–128]. As with tumorigenesis, hypoxia is necessary in early placental development. During the first trimester, placental development occurs in a low-oxygen environment due to the absence of access to maternal circulation [129]. These conditions are considered key to stimulating placental vasculogenesis. In placental fibroblasts, hypoxia upregulates both VEGF mRNA and protein [130]. One mechanism working to regulate VEGF through hypoxia is the glycoprotein Fibronectin. Fibronectin works through its high affinity integrin receptor, α5β1 to stimulate VEGF during angiogenesis of embryos as well as several tumors [131, 132]. Bovine aortic endothelial cells grown in a low pH environment to mimic hypoxia, had increased interactions between fibronectin and VEGF [133]. Additionally, low pH conditions stimulated the secretion of fibronectin into culture medium in human trophoblast cells [134]. Finally, in differentiated placental multipotent mesenchymal stromal cells (PMSCs), α5β1 has been show to interact with fibronectin to promote VEGF-A induced differentiation and migration [135].

Additionally, PlGF is also regulated by low oxygen conditions, albeit in an opposite fashion to VEGF. Human placental cells exposed to low oxygen conditions had decreased PlGF mRNA and protein [121]. Abnormal oxygen levels during early placental development are thought to lead to altered VEGF/PlGF expression leading to pre-eclampsia. For example, in the instances of pre-placental hypoxia where mother, placenta, and fetus are hypoxic (due to high altitude or anemia) there is an increase of VEGF and branched angiogenesis [136]. This phenomenon is also seen in uteroplacental hypoxia, where maternal oxygen levels are normal but there is impaired oxygen circulation throughout the placenta and fetus [137]. However, in instances of post-placental hypoxia where the mother has normal oxygen levels but the fetus is hypoxic, the placenta may become hyperoxic leading to inappropriate levels of oxygen during early development, causing increased levels of PlGF and increased non-branching angiogenesis [138]. This early onset placental hyperoxia often leads to the most severe form of pre-eclampsia, with increased adverse outcomes and fetal mortality [138].

### Genomic instability

Genomic instability is widely acknowledged as a hallmark of cancer. Ranging widely from nucleotide mutations to alternations in chromosome number or structure (known as chromosome instability), genomic instability can have major deleterious effects on normal cells [139]. However, some degree of instability appears to be tolerated by cells and has been documented in human embryos. One study analyzed blastomeres from women under 35 years of age that had undergone in vitro fertilization (IVF). Upon analysis, researchers found that 70% of all embryos had some chromosomal genomic abnormality. Additionally, only 9% of the embryos analyzed had a 100% occurrence of diploid blastomeres [140]. This suggests that genomic instability is prevalent in human embryos and potentially explains the low levels of fertility in women compared to other species. Another study analyzed levels of aneuploidy in fertilized oocytes, cleavage stage embryos, and blastocyst stage embryos. There was a large increase in aneuploidy between the fertilized oocyte stage and cleavage stage embryos. As embryos developed to the blastocyst stage, there was a significant decrease in the aneuploidy rate (83% aneuploidy in cleavage stage versus 58% in blastocyst stage). However, while there was a decrease in rates of aneuploidy, there were still high levels of overall chromosomal abnormality [141]. These data suggest that, as with tumors, for rapid placental development to occur a lapse in the cell-cycle checkpoint machinery must occur. Additionally, it has been suggested that this genomic instability actually provides an advantage for embryo implantation [142].

In addition to aneuploidy, extravillous trophoblast cells of the placenta are also polyploid [143]. These cells are analogous to murine trophoblast giant cells that are also invasive. However, rodent trophoblast giant cells have ploidy levels that can reach up to 1024 N compared to the 4–8 N recorded in extravillous trophoblast cells [144]. These cells become polyploid through a process known as endoreduplication, where cells undergo mitosis but fail to divide after DNA replication. Endoreduplication is another phenomenon that occurs in cancer to promote genomic instability [145]. It has been proposed that endoreduplication occurs during times of genomic instability to increase tissue mass while cell proliferation is decreased to prevent propagation of cells with damaged chromosomes [146]. In the placenta, extravillous trophoblast cells invade into the decidua as two different cell types, interstitial cytotrophoblast cells (iCTBs) and endovascular cytotrophoblast cells (eCTBs). The iCTBs are the cells that invade into the decidua, moving as deep as the first third of the myometrium. Once at the myometrium, these cells undergo a final step of differentiation where they undergo endoreduplication to become multinucleated [147]. Similarly to how damaged cells undergo endoreduplication to increase size, it is thought that iCTBs undergo endoreduplication to further penetrate into the myometrium of the uterus.

Finally, even with less priority attributed to cell-cycle checkpoints and DNA repair, there must be some regulation of DNA repair in the placenta for it to develop into a proper functioning organ. Our laboratory is currently focused on the regulation of DNA repair and genome stability in trophoblast cells by the tumor suppressor BRCA1. BRCA1 is a multifunctional protein involved in many different aspects of cell cycle regulation including; regulation of transcription of several proliferation factors, homologous recombination of double-stranded breaks (DSBs), cell-cycle checkpoint regulation, and chromatin remodeling [148]. BRCA1 works to repair DNA damage by acting as a scaffolding protein for other DNA repair proteins and also promotes strand-invasion by interacting with the recombinase protein, Rad51 [149, 150]. Additionally, BRCA1 forms a repressor complex with CtIP and ZNF350. This repressor complex binds to promoter regions of several oncofetal proteins to prevent transcription [151]. One oncofetal proteins target already discussed in the “cell proliferation” section is HMGA2. In addition to promoting cell proliferation, increased levels of HMGA2 causes genomic instability by preventing non-homologous end-joining as well as delaying clearance of γ-H2AX, a marker for DSBs, [152]. BRCA1^−/−^ knockout mice are embryonic lethal before gestational day 7.5 due to dramatic decreases in cell proliferation and poor differentiation of the extraembryonic tissue. These knockout embryos have a complete loss of diploid trophoblast cells with an overabundance of trophoblast giant cells [153]. Interestingly, mouse trophoblast giant cells are polyploid and are potentially accustomed to levels of genomic instability through endoreduplication, which is necessary for trophoblast giant cell function.

**Fig. 4:**
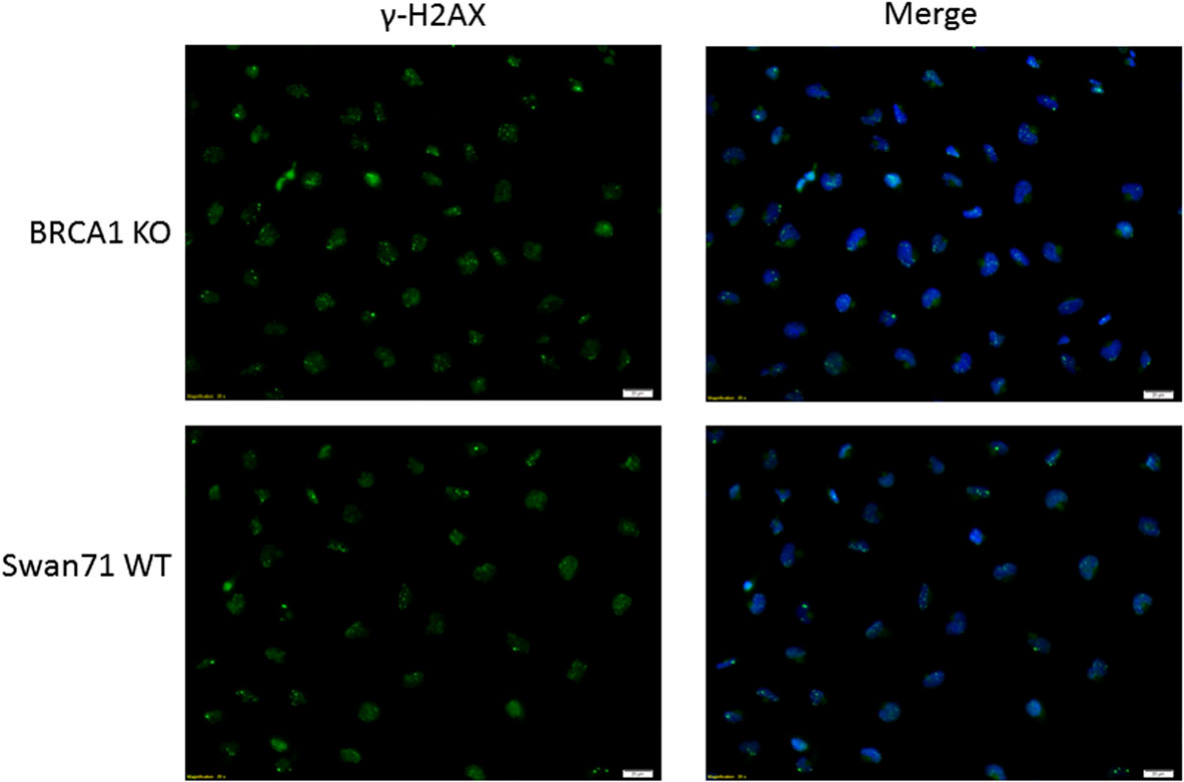
γ-H2AX in BrKO and WT Swan71 cells. Immunostaining for γ-H2AX (green) and merged with DAPI (blue) in BRCA1 knockout cells and wild-type Swan71 cells imaged at 20x magnification

Unfortunately, this question will be hard to prove using today’s current models of trophoblast cell development. Trophoblast cells derived from first trimester placentas are very difficult to obtain. Additionally these cells are hard to culture, making alternative model systems to study trophoblast development essential. Immortalized cell lines are extensively used as a model for trophoblast development and differentiation. However, these cells present their own shortcomings that make them less than ideal candidates for use. These shortcomings are especially apparent when it comes to studying DNA damage and genomic instability. For example, cytogenetic analysis of the extravillous first trimester Swan71 cell line immortalized with hTert revealed that these cells were near pentaploid in karyotype [136]. This is almost certainly due to chromosomal missegregation during mitosis, leading to a heterogeneous population of aneuploid cells. Additionally, when our lab began using this cell line to investigate BRCA1 in human trophoblast cells we found high levels of markers for DNA damage. We created a BRCA1 knockout trophoblast cell line using CRISPR-Cas9 genome editing to investigate levels of DNA damage by immunostaining for markers of double and single-stranded breaks. Surprisingly, the level of DSBs, as evidenced by immunostaining for γ-H2AX, was indistinguishable between BRCA1 knockout cells (BrKO) and wild-type Swan71 cells (Fig. 4). This high level of double-stranded breakage was confirmed using another marker for DSBs, 53BP1 (data not shown). These data corroborate the idea that immortalized cells suffer from cellular crises when cultured in vitro, resulting in microsatellite and chromosomal instability. Due to this propensity towards genomic instability in culture, immortalized cells are unlikely to provide insight into the role of genomic instability during early placental development. Additionally, this genomic instability of immortalized cells leads to a higher propensity for these cells to behave as cancer cells, no longer regulated in the controlled manner that characterizes trophoblast cells. This creates a need for a better model system to investigate the regulation of oncogenic processes during trophoblast development.

## Conclusion

While understanding the consequences of fetal growth restriction has increased exponentially over the past few decades, there is still a need to elucidate the underlying cause behind placental insufficiency during placental organogenesis. Understanding what is driving placental insufficiency during early fetal development will be essential in the development of better diagnostic and treatment tools for the prevention and treatment of IUGR. The ability of placental cells to divide rapidly, differentiate, invade and migrate into tissues, and eventually create their own vascular network makes these cells an ideal system to gain insight into cancer biology and tumor metastasis. Alternatively, as placental pathologies like intrauterine growth restriction (IUGR) and pre-eclampsia are multi-faceted disorders with no known cause, better understanding the molecular mechanisms that drive oncogenic processes will provide better insight into how the early placenta develops. Pre-eclampsia and IUGR are rarely diagnosed until after 20 weeks of gestation, significantly later than pathogenesis begins. Therefore it is critical to start thinking of oncofetal proteins in their original roles, namely as drivers of cell proliferation, differentiation, invasion, and cell survival during early embryogenesis and placental development. Studying how oncofetal proteins drive placentation is essential to facilitate the process of providing better diagnostics for earlier screenings as well as treatment, ensuring the proper care for healthier babies and happier mothers.
